# Physicochemical Properties of Flaxseed Fortified Extruded Bean Snack

**DOI:** 10.1155/2014/478018

**Published:** 2014-09-29

**Authors:** Naveen Vadukapuram, Clifford Hall, Mehmet Tulbek, Mary Niehaus

**Affiliations:** ^1^Department of Plant Science, North Dakota State University, Dept. 7670, P.O. Box 6050, Fargo, ND 58108-6050, USA; ^2^Alliance Grain Traders Inc., P.O. Box 30029, Regina, SK, Canada S4N 7K9

## Abstract

Milled flaxseed was incorporated (0–20%) into a combination of bean-corn flours and extruded in a twin screw extruder using corn curl method. Physicochemical parameters such as water activity, color, expansion ratio, bulk density, lipid content, and peroxide values of extruded snack were analyzed. Scanning electron micrographs were taken. Peroxide values and propanal contents were measured over four months of storage. Rancidity scores of extruded snack were measured using a trained panel. As expected, omega-3 fatty acids and bulk density increased with increasing flaxseed fortification levels. Extrudates with more flaxseed had decreased lightness values and expansion ratios. However, only the 15 and 20% flaxseed containing extrudates had expansion ratios that were significantly (*P* ≤ 0.05) different from the control. In general, no significant difference (*P* > 0.05) in water activity values was observed in the flaxseed fortified extrudates, except in the navy-corn based extrudates. Peroxide values increased with increased flaxseed levels and over a storage period. However, propanal values did not change significantly in the 5–10% flaxseed fortified extrudates but increased in extrudates with higher levels of flaxseed. Rancidity scores were correlated with peroxide values and did not increase significantly during storage under nitrogen flushed conditions.

## 1. Introduction

The three most physiologically important omega-3 fatty acids are alpha-linolenic acid (ALA, 18:3), eicosapentaenoic acid (EPA, 22:5), and docosahexaenoic acid (DHA, 24:6). ALA is the precursor fatty acid of EPA and DHA. ALA is an essential fatty acid that cannot be synthesized by the body [1–4]. Recently the number of foods containing omega-3 fatty acids has increased. Omega-3 fortified foods such as bread, biscuits, cakes, pasta, fruit drinks, ice cream, milk shakes, and low fat spreads have already occupied market shelves [5]. Flaxseed fortified omega-3 foods are gradually gaining importance [6]. The other reason for the rapid increase in the number of omega-3 enhanced foods is the FDA approval of a qualified health claim on conventional foods containing DHA and EPA. Furthermore, whole and milled flax were given GRAS (generally regarded as safe) status by FDA in January of 2009 [6]. The introduction of healthful ingredients into snack foods would be one way to increase the omega-3 intake by people due to the large volume of snacks consumed annually.

Plant sources for omega-3 fatty acids are flaxseed, walnut, hempseed, soybeans, canola, and rapeseed. Flaxseed (*Linum usitatissimum* L.) is a rich source of omega-3 fatty acids especially ALA. The US Institute of Medicine's Food and Nutrition Board recommends ALA intakes of 1.1 g/day and 1.6 g/day for women and men, respectively [7]. Flaxseed contains approximately 40% lipid of which 47–57% is ALA [3, 4]. Flaxseed also contains other healthful components such as dietary fiber (28%) and secoisolariciresinol diglucoside (SDG), a lignan [8]. In addition to the health benefits, flaxseed can be used to improve the nutritive value of bakery products and sensory qualities [9].

Although flaxseed has many health-promoting components, the concern over antinutrients such as cadmium and cyanogenic glycosides in flaxseed exists. North American flaxseed contains approximately 0.14 to 1.37 *μ*g/g cadmium [10] while values as high as 2.775 *μ*g/g [11] have been observed in the world flaxseed collection. The joint Food and Agriculture Organization/World Health Organization (FAO/WHO) established a provisional tolerable monthly intake (PTMI) of 25 *μ*g/kg body weight, or approximately 0.8 *μ*g/kg daily, for cadmium [12]. To put this in perspective, a 68 kg (150 lbs) would have a tolerable daily intake of approximately 54.4 *μ*g. Typical flaxseed consumption ranges from 15 to 30 g per day and thus a daily intake from cadmium would be 21 to 42 *μ*g, assuming that North American flaxseed containing the upper most cadmium level (1.4 *μ*g/g) was consumed. Another assumption is that 100% of the cadmium would be absorbed. Vanderpool and Reeves [13] reported that only 11% of the cadmium from sunflower kernels was absorbed. Therefore, similar absorption rates might be expected for flaxseed and thus the actual intake of cadmium may be lower than the estimated 21 to 42 *μ*g. Cyanogenic glycosides composition in flaxseed is approximately 300 mg/100 [14]. The release of cyanide is a concern with cyanogenic glycosides during digestion. However, cyanogenic glycosides can be reduced through appropriate high temperature processes [15]. In addition, 84–89% reductions in cyanogenic glycosides have been reported in extruded flaxseed [16, 17].

There is an inverse connection between ALA content and susceptibility of lipid oxidation [18]. The high level of ALA (50–57%) in flaxseed oil makes it more susceptible to oxidation than other oils, which have lower levels of unsaturation. Other oils containing long chain omega-3 fatty acids, for example, DHA and EPA, are even more prone to oxidation. ALA stability over DHA and EPA makes it an ideal option for food fortification.

Omega-3 fatty acids degrade in the presence of heat, light, and oxygen. Thus, developing foods containing omega-3 fatty acids is a challenge [19]. The primary oxidation products of omega-3 fatty acids are hydroperoxides. Aldehydes and ketones are secondary oxidation products that arise from hydroperoxide decomposition. The major degradation product of omega-3 fatty acid, propanal, an aldehyde, is used as an indicator of omega-3 oxidation [20].

Only a few reports are available regarding the extrusion of flaxseed in a cereal or pulse base [21–23]. However, shelf lives were not included in previous studies. Therefore, the current project was undertaken due to the lack of knowledge regarding the shelf life stability of extruded bean snacks fortified with flaxseed. Bean flours were chosen as a carrier of flaxseed due to nutritional and functional characteristics of beans in extruded foods [24, 25]. The objective of this research was to determine the effect of flaxseed on the physicochemical, sensory, and shelf life properties of flaxseed fortified extrudates.

## 2. Materials and Methods

### 2.1. Flour Blends

Navy and pinto bean flours were obtained from Heartland Ingredients (Crookston, MN). Corn flour was obtained from Cargill Horizon Milling (Wayzata, MN). Cold-milled yellow flaxseed was obtained from Puretec International Inc. (Valley City, ND) two weeks prior to extrusion. Blends of navy and corn flours (50 : 50 wt/wt) and pinto and corn flours (50 : 50 wt/wt) were mixed in a twin shell-type mixer (45.3 kg capacity) for 10 min. To each of these bean-corn flours, different levels (0, 5, 10, 15, and 20%) of cold milled flaxseed (595 *μ*m particle size) were added and mixed using a twin shell-type blender for 20 minutes. Five combinations of flaxseed-bean-corn mixes were made for each of the bean flours, resulting in a total of 10 treatments. The blending was completed 3 separate times (i.e., replication) for each of the flaxseed levels.

### 2.2. Extrusion and Sample Preparation

Combinations (navy-corn-flax and pinto-corn-flax) were extruded using a twin screw extruder (Wenger TX 52, Atkinson, KS). Standard puffed corn curl method (high temperature and short time) was employed according to standard processing and screw design provided by Wenger. A die with 1.5 mm diameter was used during extrusion. A water feed rate was adjusted to 0.128 kg/min and screw rpm was set at 310 rpm. Material feed rate was adjusted at 10 rpm (approximately 110 lb/hr). The zone (1 to 7) temperatures were 36, 37, 41, 53, 85, 106, and 110°C, respectively. Extrudates were cooled to room temperature, subdivided into 150 g batches, and packed into 15 cm × 25 cm metallized barrier pouches (2.5 mil; Associated Bags Company, Milwaukee, WI) sealed under nitrogen. Packaged samples for shelf life evaluations were stored at 22 ± 2°C. Time zero samples were stored in sealed bags in a freezer until analytical testing could be conducted on the extrudates. The analytical test completed on the frozen sample included all physical test methods (Section 2.3), lipid and oxidation measurements (Section 2.4), and sensory evaluation (Section 2.5). The analytical tests completed during the shelf life evaluation included oxidation measurements (Section 2.4) and sensory evaluation (Section 2.5).

### 2.3. Physical Parameters

Modified methods [23, 26] were used to analyze physical parameters (i.e., texture, color, expansion ration, bulk density, and water activity). Texture was analyzed using a texture analyzer (Brookfield LFRA, Middleboro, MA). A cutting wire of length 40 mm and 0.33 mm thickness (Model TA26) and test speed of 1 mm/sec were used to complete texture analysis. Color values of ground samples were analyzed using a Hunter Minolta colorimeter (Chroma Meter CR-310, Ramsey, NJ, USA). Water activity values were measured using water activity meter (Aqua Lab Series 3TE, Decagon Devices, Pullman, WA). Scanning electron microscopy (SEM) analysis of the finished product was determined at ×10 (8 Kv) and ×550 (8 Kv).

### 2.4. Lipid and Oxidation Measures

Extrudates (75 g) were ground using a Retsch ZM 200 ultracentrifugal mill (Haan, Germany) with a 1 mm ring sieve. Lipid content of ground samples was determined using a 16 hr soxhlet extraction method [20]. However, oil for peroxide value (PV) was extracted at room temperature using chloroform : methanol (2 : 1 v/v) for three hours [20]. The solvent to ground extrudate ratio was 10 : 1. The lower temperature room extraction was done to prevent potential breakdown of the hydroperoxides. Peroxide values (PV) on the extracted oil were analyzed using the method of Crowe and White [27].

Headspace solid phase microextraction (HS-SPME) was used to identify propanal, which is formed during the oxidation of ALA [20]. Propanal was analyzed using gas chromatograph, GC5890 series II (Hewlett Packard, Palo Alto, CA). The SPME filament (divinylbenzene/carboxen/polydimethylsiloxane) (Supelco, Bellefonte, PA) of 30/50 *μ*m thickness was used. A Zebron (Phenomenex, USA) capillary GC column (60 m length, 0.25 mm i.d., and 0.20 *μ*m film thickness) was used to separate propanal following our previous protocol [20].

The ALA content was determined using the fatty acid profile method [28]. A GC 5890 series II equipped with a flame ionization detector and a SP2330 fused silica capillary column (30 m length, 0.25 mm i.d., and 0.20 *μ*m film thickness) (Supelco, Bellefonte, PA) was used to separate the fatty acids. The ALA content was reported on mg/100 g of extrudate basis.

### 2.5. Sensory Evaluation

A trained sensory panel of seven members was employed to assess the rancidity of the extrudates. Four of the seven members had previous experience evaluating rancidity in other flaxseed fortified products. Six females and one male participated in the sensory panel. Ages of the panelists ranged from 20 to 55. Prior to the extrudate evaluation, the panel was trained for a total of six hours (1.5 h × 4 sessions) using freshly made bean-corn extrudates (no flaxseed) spiked with various levels of rancid (PV = 35 meq/kg) flaxseed oil. The panelists defined the term rancid as painty after a combination of smelling and orally tasting the sample. The scale used during training was broken into segments indicating the intensity of the painty characteristic as not detectable (0 cm), barely detectable (1.31 cm), very little (2.08 cm), moderate (3.7 cm), strong (6.95 cm), very strong (9.5 cm), and strongly imaginable (16.6 cm). A 16.6 cm unstructured line scale was used to assess the paintiness (i.e., rancidity) during the 4-month shelf life study. To reduce fatigue, the navy-based extrudates and pinto-based extrudates were run on separate days and only the 0, 10, and 20% extrudates from all three replications, based on original processing replications, were evaluated. A blind moderate positive control was used during the sensory evaluation as a means to assess whether panelist retained their ability to differentiate the painty characteristic. In total, panelists evaluated 10 samples for paintiness during the monthly session.

### 2.6. Statistical Design

Arandomized complete block design was used. Each set of treatments was considered as separate experiment. Experiments were replicated three times based on three different lots of raw material (i.e., flaxseed). Results were analyzed using ANOVA at 95% level of significance (*P* ≤ 0.05). Pearson's correlation coefficients between the treatment means were determined using SAS software.

## 3. Results and Discussion

### 3.1. Physical Parameters

#### 3.1.1. Color

**Figure 1:**
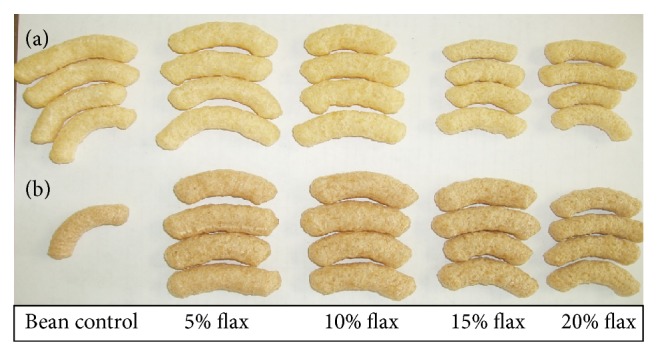
Extrudates obtained from combinations of navy (a) or pinto (b) bean and corn flours containing flaxseed (5–20%).

The color of the pinto flours contributed to enhanced darkness (Table 1 and Figure 1). Increased flaxseed concentration in both flours reduced the *L* values of the extrudates, suggesting that flaxseed contributed to the darkening of extrudates and is in agreement with results reported by Wu et al. [23]. However, color darkening (i.e., decreasing *L* values) of the extrudates containing 10 to 20% flaxseed was not significant (*P* > 0.05). Furthermore, with a few exceptions, no significant difference (*P* > 0.05) was observed in redness (*a*) and yellowness values (*b*) in both pinto and navy extrudates containing increased flaxseed levels (Table 1 and Figure 1).

#### 3.1.2. Water Activity, Bulk Density, Expansion Ratio, and SEM Values

Water activity values (Table 2) increased with increasing flaxseed percentage in navy-corn extrudates. The increase was significant (*P* ≤ 0.05) for the extrudates made with navy-corn and all levels of flaxseed. In contrast, water activity was not affected by flaxseed fortification in bean extrudates containing pinto bean flours. The range (0.4–0.6) in water activity values indicates the potential for a stable shelf life of the products. Lipids are most stable to oxidation when water activity is within the range of 0.3 to 0.6 [29]. Only extrudates containing 15% and 20% flaxseed and navy bean flour had water activities outside the 0.3 to 0.6 range. All water activities under 0.75 values are considered acceptable in preventing microbial growth [29].

In general, bulk density values of extrudates containing pinto flours and navy flours were not significantly different (*P* > 0.05) for extrudates with 5% and 10% levels of flaxseed (Table 2). However, the bulk density values were significantly different (*P* ≤ 0.05) for extrudates containing 15% and 20% flaxseed compared to the control and lower flaxseed-containing extrudates (Table 2). Ahmed [30] also reported increased bulk density of flaxseed containing corn extrudates obtained via single screw extrusion. High protein and high fiber materials added to starch-based flours tend to increase the density of extrudates [31]. The increased bulk density values may be due to the high fiber and high protein components in bean flours and high fiber content in flaxseed interfering with the starch expansion [32]. Bulk density values and expansion values were negatively correlated in navy-corn (*r* = −0.644, *P* < 0.05) and pinto-corn (*r* = −0.80, *P* < 0.05) extrudates.

Degree of expansion affects density, fragility, and overall texture of extruded products [25]. Expansion values were not significantly (*P* > 0.05) different between 5% and 10% flaxseed-containing navy-corn extrudates and 5%, 10%, and 15% flaxseed containing pinto-corn extrudates (Table 2). Starch is the primary cause for expansion [25]. Expansion values of pinto-corn extrudates were found to be slightly higher than those of the navy extrudates, which was likely due to higher starch content in pinto beans. The addition of protein tends to decrease expansion of extruded snacks [25]. Furthermore, the high fiber of flaxseed [8] likely contributed to the reduction in expansion because the high flaxseed (20%) extrudates had the lowest expansion values. Fiber has a tendency to rupture cell walls and promotes breakage of air cells during extrusion, which prevents matrices from expanding [32].

**Figure 2:**
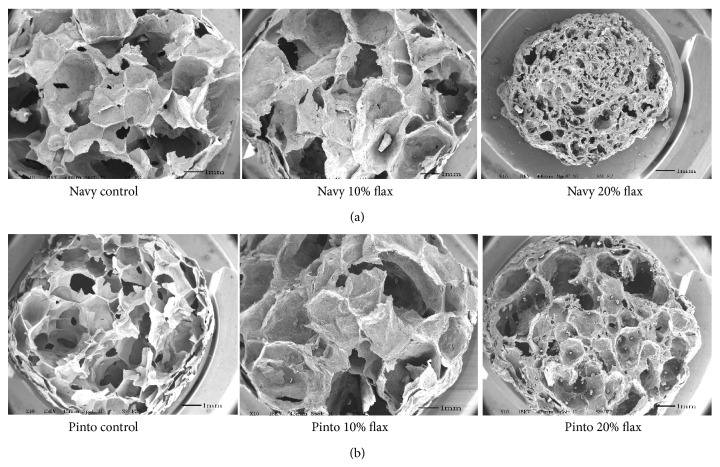
Scanning electron micrographs (×550 magnification) of extrudates obtained from combinations of bean flour and corn (i.e., navy or pinto control) and either 10 or 20% flaxseed.

Scanning electron micrographs (Figure 2) further indicated that the structure was more compact with increasing levels of flaxseed. However, scanning electron micrographs of extrudates containing 5 and 10% flaxseed were not different from those of the control samples; thus only the 10% is shown. The 20% flaxseed fortification clearly affected the structure of the extrudates. Flaxseed addition at higher levels (20%) affected the structure of the navy-corn extrudates to a larger extent compared to that of the pinto-corn system (Figure 2). The combination of protein and fiber, along with a reduction in starch content, likely led to the more compact structure of the 20% flaxseed extrudates.

### 3.2. Chemical Parameters

#### 3.2.1. Lipid and ALA Contents

Lipid was extracted from the ground flaxseed, the dry mix before extrusion, and the ground extrudates after extrusion. Lipid content of the flaxseed was approximately 44% and contributed to a significant (*P* ≤ 0.05) increase in oil content with increasing flaxseed levels in both navy and pinto extrudates (Table 3). Lipid content of the finished product indicated that either the extrudates lost oil during the extrusion process or the lipid became bound to starch or protein components. The lipid content before and after extrusion indicated that only about 50% of the estimated lipids were extracted from the extrudates. We have observed that soxhlet extraction with hexane can recover about half of the lipid from extruded products (unpublished data). In addition to extraction issues, the loss of oil during the extrusion process was observed for the 20% flaxseed containing extrudates. This suggests that the high pressure was pressing some of the oil from the flaxseed during extrusion. However, the oil content in extrudates with lower levels of flaxseed and the control extrudates may involve the formation of lipid-starch or lipid-protein complexes [33–35] because no visible oil was observed on the extrudates or being expelled during the extrusion process.

The ALA levels increased significantly (*P* ≤ 0.05) with the increased flaxseed level in extrudates (Table 4). In addition, the percentage of ALA did not change during the four-month storage (data not reported). Imran and Anjum [36] reported that, under optimum extrusion conditions, 99.9% of the ALA was retained after extrusion of flaxseed meal. This suggests that the ALA did not degrade during extrusion and supports the theory that the lower ALA content in the bean-corn-flaxseed extrudates was a result of a binding of the lipid with other components [35]. ALA values on a per serving basis indicate that the increase of ALA values was proportional to the increase in flaxseed fortification (Table 4). The increased lipid and ALA levels likely contributed to the observed textural, bulk density, and rancidity characteristics.

#### 3.2.2. Oxidation and Rancidity

The PV of the pinto and navy extrudates indicated that the rancidity of extrudates was affected by the increase in flaxseed level (Table 5). Peroxide values of 5 and 10% flaxseed extrudates at time zero were not significantly different (*P* > 0.05) compared to the navy corn extrudates. However, the PV in the 15 and 20% flaxseed containing navy-corn extrudate were significantly (*P* ≤ 0.05) different from the control and 5% flaxseed treatments at time zero while no difference in PV was observed between the extrudates containing 10% and 15% flaxseed. Similar results were observed in the pinto-corn extrudates (Table 5). The 20% flaxseed containing extrudates had PV that were significantly higher than the control and 5% flaxseed extrudates, but not significantly higher than the 10% flaxseed containing extrudates at the four-month storage time. The higher PV in the 20% extrudates is probably due to the increased availability of lipid on the surface of the extrudate, which can more readily oxidize.

Although the PV was relatively low, the PV and sensory scores were well correlated (0.99 and 0.92, resp.; *P* ≤ 0.01) in pinto extrudates at time zero (*T* = 0) and four months (*T* = 4). Similar correlations for the navy extrudates (0.99 and 0.98 at *T* = 0 and *T* = 4, resp.; *P* ≤ 0.01) were observed. However, the 5% and 15% flaxseed extrudates were not included in the correlation analysis since these extrudates were not included in the sensory evaluation. The 5% flaxseed extrudates had similar PV compared to the control (Table 5) while the 15% flaxseed extrudates were similar to the 20% flaxseed extrudates. Therefore, 5% and 15% flaxseed extrudates were dropped from the sensory evaluation because of the similarity in rancidity indicators and the need to reduce panelists fatigue due to the large number (10 per session) of samples being evaluated.

No propanal, the major aldehyde produced during degradation of ALA, was found in zero time (Table 6) or the two-month-old extrudates (data not reported). A small increase in propanal was observed in four-month-old samples (Table 6). The possible reason for the increased oxidation might be related to the surface lipids. Some of the lipid may have been expelled from the protective coating of a starch-protein matrix that forms during extrusion processing. As a result, the lipid may have been more accessible to oxygen and thus oxidation. The data at four months suggests that the flaxseed addition did affect propanal formation. Propanal values were not significantly different between the control and 5% flaxseed containing extrudates in both pinto and navy extrudates, although the values increased with increasing flaxseed levels. However, the reduced propanal level (Table 6) observed in the 20% flaxseed extrudate, compared to the 15% flaxseed sample, may be due to the more compact structure of the 20% flaxseed extrudates (Figure 2). High correlations (0.99 and 0.91; *P* ≤ 0.01) between propanal content and sensory scores of the 4-month stored navy and pinto extrudates, respectively, were observed. Correlations observed suggest that as oxidation markers (i.e., PV and propanal) increased, so did the painty characteristic of the extrudates.

The extrudate containing 20% flaxseed did have the highest rancidity score (Table 5) indicating oxidation. In general, increasing flaxseed concentrations increased the rancidity scores in samples stored for four months. However, no differences in rancidity scores were observed in the time zero samples. This indicated that the extrusion process did not promote the oxidation of flaxseed during extrusion. The higher rancidity scores observed by the sensory panel were likely due to the increased oxidation that occurred in the high lipid samples during storage, even though the samples were stored in a nitrogen flushed package. Furthermore, small increases in rancidity of the 20% flaxseed extrudates may be related to ALA content, which was the highest in the 20% flaxseed extrudates. A limitation of the study might be that oxidation was not measured more frequently during the storage study. The samples were stored under a nitrogen flush metalized package typical of the snack foods; thus, the low level of oxidation was anticipated. Although not reported in the data in this paper, we did observe that the 20% flaxseed extrudates left open to air and under fluorescent light reached PV of approximately 20 meq/kg in as little as 5 days after extrusion. Therefore, the need for packaging under inert environments and light impermeable packaging was evident.

## 4. Conclusion

Milled flaxseed addition significantly increased water activity and omega-3 fatty acid content of extrudates. The 5 and 10% milled flaxseed addition did not significantly affect lipid oxidation and sensory attributes. Interactions between starch, protein, and lipids may have contributed to the stability of extrudates. Extrudates from bean and corn flour combinations with 5 and 10% flaxseed had good quality and stable shelf life. In addition, consuming 1 oz (28 g) of the 10% fortified extrudate provides a significant amount (i.e., approximately 33%) of the recommended daily ALA intake. These levels of flaxseed are recommended for the creation of a gluten-free extruded healthy snack.

## Figures and Tables

**Table 1:** Mean color values of navy-corn and pinto-corn extrudates.

Treatment	Navy-corn			Pinto-corn		
	*L*	*a*	*b*	*L*	*a*	*b*
Control	78.1^bc^	3.16^a^	28.75^a^	71.16^d^	1.29^d^	22.82^a^
5% flax	79.3^c^	3.96^b^	29.43^ab^	70.24^bc^	1.12^cd^	23.07^a^
10% flax	77.48^ab^	3.81^ab^	30.19^b^	69.68^ab^	0.96^c^	22.96^a^
15% flax	77.42^ab^	3.95^b^	29.4^ab^	68.95^a^	0.64^b^	22.84^a^
20% flax	76.35^a^	3.55^ab^	28.72^a^	69.78^ab^	0.12^a^	22.93^a^

*L*: lightness; *a*: (+) red, (−) green; *b*: (+) yellow, (−) blue. Means in column with the same letter are not significantly (*P* > 0.05) different.

**Table 2:** Mean water activity, bulk density, and expansion ratio values of navy-corn and pinto-corn extrudates.

Treatment	Navy-corn			Pinto-corn		
	Water activity	Bulk density (g/cm^3^)	Expansion ratio	Water activity	Bulk density (g/cm^3^)	Expansion ratio
Control	0.48^a^	69.1^a^	7.8^b^	0.52^a^	71.4^a^	8.0^b^
5% flax	0.54^b^	72.1^a^	7.9^b^	0.54^a^	77.9^a^	8.0^b^
10% flax	0.60^bc^	87.1^a^	8.5^b^	0.54^a^	78.2^a^	8.1^b^
15% flax	0.64^c^	126.4^b^	7.7^ab^	0.57^a^	98.5^b^	7.5^b^
20% flax	0.69^d^	177.4^c^	6.9^a^	0.57^a^	132.6^c^	6.2^a^

Means in column with the same letter are not significantly (*P* > 0.05) different.

**Table 3:** Lipid content of the bean-corn flour mix and extrudates.

Treatment	Navy-corn		Pinto-corn	
	Lipid % before extrusion	Lipid % after extrusion	Lipid % before extrusion	Lipid % after extrusion
Control	1.76^aA^	0.93^aB^	1.67^aA^	0.41^aB^
5% flax	4.18^bA^	1.09^aB^	3.92^bA^	1.27^bB^
10% flax	6.21^cA^	2.78^bB^	6.11^cA^	2.37^cB^
15% flax	8.41^dA^	4.26^cB^	8.22^dA^	3.92^dB^
20% flax	10.61^eA^	5.85^dB^	10.54^eA^	5.72^eB^

Means in column with the same lowercase letter are not significantly (*P* > 0.05) different. Means in row with the same uppercase letter are not significantly (*P* > 0.05) different.

**Table 4:** *α*-Linolenic acid (ALA) in the bean-corn extrudates.

Treatment	Navy-corn		Pinto-corn	
	mg ALA/100 g	mg ALA/serving	mg ALA/100 g	mg ALA/serving
Control	386^a^	108^a^	126^a^	35^a^
5% flax	479^b^	134^a^	555^b^	155^b^
10% flax	1359^c^	381^b^	1146^c^	321^c^
15% flax	2136^d^	598^c^	2259^d^	632^d^
20% flax	3071^e^	860^d^	3772^e^	1056^e^

Serving is based on 1 oz or 28 g. Means in column with the same lowercase letter are not significantly (*P* > 0.05) different.

**Table 5:** Peroxide values and rancidity scores of navy-corn and pinto-corn extrudates at different time periods.

Treatment	Navy-corn				Pinto-corn			
	Peroxide values		Sensory scores		Peroxide values		Sensory scores	
	*T* = 0	*T* = 4	*T* = 0	*T* = 4	*T* = 0	*T* = 4	*T* = 0	*T* = 4
Control	2.9^aA^	3.4^aB^	2.5^aA^	3.5^aB^	3.0^aA^	3.2^aA^	2.4^aA^	2.5^aA^
5% flax	3.1^aA^	3.6^aB^	ND	ND	3.0^aA^	3.5^abB^	ND	ND
10% flax	3.3^abA^	3.7^abA^	2.6^aA^	4.5^abB^	3.1^aA^	3.6^abcA^	2.4^aA^	4.4^abA^
15% flax	3.6^bA^	4.0^bA^	ND	ND	3.2^abA^	3.8^bcB^	ND	ND
20% flax	3.8^bA^	4.2^bA^	2.8^aA^	5.6^bB^	3.5^bA^	4.2^cB^	3.4^aA^	5.0^bB^

*T* indicates time in months. Means in column with the same lowercase letter are not significantly (*P* > 0.05) different. Means in row with the same uppercase letter are not significantly (*P* > 0.05) different. ND indicates not determined.

**Table 6:** Propanal values of navy-corn and pinto-corn extrudates at different time periods.

Treatment	Navy-corn		Pinto-corn	
	Propanal (ppb)		Propanal (ppb)	
	*T* = 0	*T* = 4	*T* = 0	*T* = 4
Control	ND	45.3^a^	ND	59^a^
5% flax	ND	58.7^ab^	ND	64.3^ab^
10% flax	ND	79.3^bc^	ND	85.7^abc^
15% flax	ND	112.3^d^	ND	108^d^
20% flax	ND	101^cd^	ND	106^cd^

*T* indicates time in months. Values in column having the same letter are not significantly (*P* > 0.05) different. ND indicates no propanal detected (i.e., 0).
